# Plasma Surface Engineering of Natural and Sustainable Polymeric Derivatives and Their Potential Applications

**DOI:** 10.3390/polym15020400

**Published:** 2023-01-12

**Authors:** Renjith Rajan Pillai, Vinoy Thomas

**Affiliations:** Department of Material Science and Engineering, The University of Alabama at Birmingham, Birmingham, AL 35294, USA

**Keywords:** applications, biomedical, heavy metal remediation, packaging material, sensor development natural fibers, plasma surface modification, sustainable polymers, surface treatment, water purification

## Abstract

**Highlights:**

- The article reviewed plasma research on natural fiber functionalization and sustainable polymeric derivatives in the last two decades.
- The article highlighted various plasma processes: functionalization, ablation, ion implantation, grafting, and polymerization, with special emphasis on natural fiber materials.
- The review article focused on plasma-modified sustainable materials’ application in heavy metal remediation, water purification, biomedical applications, packaging materials, and sensor applications.
- Plasma processing employed fortuitously to alter the natural fibers and sustainable polymeric derivatives for potential applications reducing the environmentally hazardous chemicals.
- The revamped surface properties such as hydrophilicity, adhesion, and surface energy of the materials by dint of plasma processing ensures wide applicability of the sustainable polymeric materials.

**Abstract:**

Recently, natural as well as synthetic polymers have been receiving significant attention as candidates to replace non-renewable materials. With the exponential developments in the world each day, the collateral damage to the environment is incessant. Increased demands for reducing pollution and energy consumption are the driving force behind the research related to surface-modified natural fibers (NFs), polymers, and various derivatives of them such as natural-fiber-reinforced polymer composites. Natural fibers have received special attention for industrial applications due to their favorable characteristics, such as low cost, abundance, light weight, and biodegradable nature. Even though NFs offer many potential applications, they still face some challenges in terms of durability, strength, and processing. Many of these have been addressed by various surface modification methodologies and compositing with polymers. Among different surface treatment strategies, low-temperature plasma (LTP) surface treatment has recently received special attention for tailoring surface properties of different materials, including NFs and synthetic polymers, without affecting any of the bulk properties of these materials. Hence, it is very important to get an overview of the latest developments in this field. The present article attempts to give an overview of different materials such as NFs, synthetic polymers, and composites. Special attention was placed on the low-temperature plasma-based surface engineering of these materials for diverse applications, which include but are not limited to environmental remediation, packaging, biomedical devices, and sensor development.

## 1. Introduction

Growing demand in the field of renewable and environmentally friendly materials has generated interest in current research focused on NFs, polymers, and their derivatives. There are historic indications, through the investigation of wool and flax fabric at the excavation sites of nation lake dwellers in the 6th and 7th centuries BCE and in prehistoric times, that many vegetable fibers were also used as textiles [1]. Hemp fibers originated from Southeast Asia and further extended to China, where cultivation was reported to date to BCE 4500. Linen weaving and spinning were developed in Egypt in BCE 3400, and cotton spinning was started in India in BCE 3000. Sericulture (row silk production through the cultivation of silkworms) was invented and developed in China in BCE 2640, and later, silk manufacturing received attention all around the world. These historical facts are suggestive of the role of natural fibers in human life from prehistorical times. Fibrous materials, mainly cellulosic types like cotton, grains, wood, and straw, were often used in industrial applications and textiles. One of their most important uses was as a reinforcing material in polymer composites due to their abundance, biodegradability, cost-effectiveness, renewability, pliability, elasticity, less abrasive nature, etc. [2,3,4]. The role of NFs increased rapidly as time progressed, and now in the modern world, they have found applications in different sectors of life [5]. The uses of NFs were confined mostly to textiles but now extend to various interphases, composites, recycle strands, biodegradable matrices, micromechanics, biobased polymers, structural materials, and so on [6,7,8].

Like NFs, another class of important materials that play a significant role in applied engineering and technological advancements is synthetic polymer fibers. One of the major drawbacks associated with the usage of polymers is waste management [9,10]. As polymer chemistry has led to various synthetic polymers that the global economy depends on, it can undoubtedly contribute vital solutions to address the current polymer waste challenge. Apart from synthesizing various polymers, it could also contribute to the development of innovative polymers having inherent recyclability potential which further enhances the material properties and performance. NF-reinforced polymer composites (NFRPCs), plasma surface modification (PSM) of natural polymers, synthetic polymers, their derivatives, etc., were the major inventions that met these requirements. These can be termed sustainable polymers, or a class of materials that exhibit a closed-loop lifecycle. They were further redefined as materials derived or combined with renewable feedstocks that are safe in both production and use and can be recycled or disposed of in an environmentally innocuous manner. Hence, it is very important to get a comprehensive overview of the recent developments taking place in this field. Inspired by this, in this extensive review, we discussed natural and synthetic polymers, their drawbacks, and innovative applications via PSM that have taken place in the last two decades.

### 1.1. Natural Fibers

**Figure 1:**
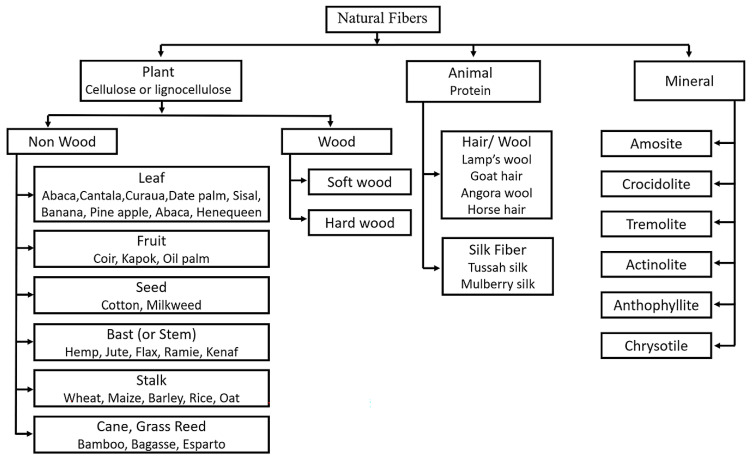
An overview of the classification of NFs.

NF sources exist all around the world and are primarily classified by their origins, such as plants, animals, and minerals, as shown in Figure 1 [11,12,13]. The extraction methods and different processing techniques determine the quality of the fibers. Fibers of plant and animal origin have been most widely used, while mineral-derived fibers have hardly ever been discussed; they are beyond the scope of this review. Plant-based (cellulosic or lignocellulosic) fibers are classified as wood and non-wood. Among the non-wood fibers, parts of plants such as leaves, fruit, seed, stem, stalk, and cane, are utilized to extract fibers for different uses with and without further processing. In ancient times, these were mainly used for fabric and packaging applications. Animal (protein)-based fibers are mainly of two types, hair or wool and silk fibers [14]. Animals’ hair has significantly been used for winter wear, most commonly lamb, goat, angora, and horse [15].

The major challenges with NFs are their high moisture absorption, poor thermal stability, quality variations, low hydrophobic polymer matrix compatibility, and color variations with sunlight [16,17]. The presence of polar groups imparts a hygroscopic nature to most NFs; hence, mechanical properties such as strength and stiffness vary [18]. NFs are not that effective in stress transfer via adhesion with polar matrix materials. The physical and mechanical properties can be explained relative to the size and shape of cellulose molecules and different bonds, the interaction of the non-cellulose components of the fibers, and the fibrillar arrangements.

Drawbacks with cellulosic fibers:

1. Improvement of properties via spinning is limited by poor solubility in general solvents.
2. They have poor crease resistance, due to which garments with cellulosic fibers become crumpled during wear.
3. Poor thermo-plasticity limits the use of garments.
4. Less dimensional stability leads to distortion of garments during ironing and laundering.

### 1.2. Synthetic Polymers

Synthetic polymers have contributed to the development of durable, flexible, and wear-resistant polymeric systems [19,20,21]. Bakelite was the first synthetic polymer, invented in 1907, and the first modern plastic was polyvinyl chloride (PVC), made usable in the 1920s with improved plasticity using additives [22]. Synthetic polymers are a family of materials that are classified as thermoplastic and thermosets based on their ability to change their solidity by the application of heat in both directions [23]. Thermoplastics such as PVC, polyethylene (PE), polypropylene (PP), polyethylene terephthalate (PET), polyamide (PA), and polystyrene (PS) can be remolded by applying heat; thus, they are recyclable. On the other hand, thermosets such as polyurethane (PU), unsaturated polyester, silicone, and epoxy resins (melamine, acrylic, and phenolic resins) undergo chemical changes with heating, forming an irreversible 3D-crosslinked structure. They cannot be remelted and reformed; hence, they are non-recyclable. Both thermoplastics and thermosets are known for compositing with glass and carbon [24].

Another way of classifying synthetic polymers is based on the carbon source, a fossil or recent biomass [25]; the major categories are biobased plastic and fossil-based (petroleum-based) plastic. Most of the current fossil-based plastics are non-biodegradable. The common notion that all biobased plastics are biodegradable is false. Most biobased plastics are chemically identical to one of the fossil-based polymers, such as PET or PE, and thus share their biodegradable nature. The Coca-Cola company developed a prominent PlantBottle material, consisting of 30% mono ethylene glycol (MEG) and 70% purified terephthalic acid [26]. The MEG for the actual PlantBottle material was made from Brazilian sugarcane, whereas the terephthalic acid was fossil-based. Coca-Cola announced a 100% biobased PlantBottle in 2015 [27].

Synthetic polymers are the most viable alternative to many conventional materials such as metals and wood. This is because of their intact post-treatment mechanical strength and resistance to thermal deterioration and solvent treatments. Acrylonitrile butadiene styrene (ABS) is a strong, hard, and flexible synthetic polymer found in objects as diverse as camera cases and car bumpers. Some more are PS, which can be easily molded into different shapes such as plastic forks, plates, etc., and polystyrene foam (Styrofoam), used as a thermal insulator and popular as beverage containers in restaurants. Synthetic polymers are an integral part of the modern world that have made life more convenient and easier in different ways. However, this does not mean that synthetic polymers are free of challenges.

The raw materials for synthetic polymers are not limitless, and their disposal has led to environmental pollution. The surface properties of synthetic polymers limit their use in many applications; therefore, surface modification is necessary to improve and suppress various properties to meet the requirements [28]. For example, hydroxyl groups on the surface impart hydrophilicity, which is not an inherent property of all polymeric surfaces. So, the intrinsic weaknesses of the inherently non-conformant surfaces were modified to facilitate various industrial applications. Another major challenge with synthetic polymers is waste management [29]. The lack of reusability in certain cases, inherent nondegradable nature, and limited awareness among users have resulted in environmental pollution. Researchers have focused on solutions for the challenges offered by synthetic polymers [30,31,32].

### 1.3. Natural-Fiber-Reinforced Polymer Composites (NFRPCs)

It is clear from the previous discussion that neither NFs nor synthetic polymers can address different issues, such as recyclability, for different industrial applications. This has inspired researchers to explore a combination approach where NFs and synthetic polymers were combined to design NFRPCs. This strategy can significantly improve the application potential of these materials. NFs can be converted into a composite by mixing them with suitable materials based on the applications and prerequisites [33,34]. In recent times, NFRPCs found roles in different engineering applications because of their recyclability and sustainability as compared to raw as well as processed NFs. The central point that upholds their use in structural applications was their ability to offer lightweight parts without compromising strength compared to manufactured and metallic materials. NFRPCs emerged as an efficient substitute for ceramics and metals in automotive, aircraft, electronics, sports, goods, and marine industries [35]. Table 1 provides information regarding the large-scale production of NFs which have been commercially used in composites across the world [4].

Considering their inexhaustible nature as well as their good potential for some degree of recyclability and biodegradability, NFRPCs has received consideration for both essential exploration and mechanical applications. One of the significant necessities of the current scenario is eco-friendly materials and strategies without compromising the prerequisites. Scientists have grown new fiber-assembling patterns for composite materials utilizing NFs like flax, cotton, hemp, sisal, jute, kenaf, banana, pineapple, bamboo, wood, and so forth [37]. There are multiple aspects of composite materials that can influence the level of performance/activities, of which the following are a few examples.

(i) Fiber orientation [38];
(ii) Fiber strength [39];
(iii) Various physical properties of fibers [40];
(iv) Interfacial adhesion between fibers [41].

Germany is the leading nation in the use of NFRPCs due to its automobile industry. Automobile manufacturers such as BMW, Audi, Volkswagen, and Mercedes started using NFRPCs for parts; this later earned worldwide attention [42]. Mercedes S-Class cars started using NFRPCs for the inner door panel in 1999, which was 35% baypreg-F, a semi-rigid elastomer made of bay, and 65% sisal, flax, and hemp. From this, it was evident that NFRPCs were developed and used for environmental needs and to generate global awareness [43]. Hybrid composites were made of different fibers so that the drawback of one can be balanced by the other; they are proven alternatives for existing materials [44,45]. The application of NFRPCs in automobiles includes door and instrument panels, arm- and headrests, parcel shelves, seats [46], bumpers (glass/kenaf epoxy composite) [47], underfloor protection of passenger cars (banana-fiber-reinforced composites) [48], indicator coverings, and nameplates (sisal and roselle hybrid composites) [49], decks, window frames, and molded panel components (plastic/wood fiber composites) [50]. Hydrophilicity was the major challenge faced by NFs as a reinforcing material in synthetic polymers [51]. In cellulose-based composites, a high aspect ratio (length/width) played a vital role, as it gave an idea of the mechanical properties. NFs were selected based on fiber strength, dimensions, defects, variability, and crystallinity. Table 2 discusses the reported works on NFRPCs.

## 2. Requirements and Methods of Polymer Modifications

The choice of polymers for different applications largely depends on the surface as well as the bulk properties. There have been several surface-tailored natural and synthetic polymers that resulted in added mechanical properties that improve the strength and structure [111]. Contrary to brittle synthetic polymers, NFs possess a high degree of elasticity and are less likely to fracture (having a high aspect ratio) during fabrication processes, including extrusion and injection molding. The poor interfacial attraction between the cellulose filaments and thermoplastic matrix limits the thermal stability and mechanical strength of composites [112,113]. Efficient composites were accomplished by either surface modification of fibers to make them more viable with the matrix or by using coupling agents that stick well to both the matrix and fibers [114]. In general, fiber surface modification is important for improving fiber–matrix adhesion and reducing the moisture absorption by NFs to improve thermal degradation, flammability, and mechanical properties [115]. Modifications can also be done by physical, chemical, and biological methods for property enhancement like improved mechanical and composite properties, increased tensile strength, and considerably higher polar components with free surface energy [116,117].

### 2.1. Chemical Techniques

Chemical modification offers improved mechanical properties [118] and compatibility between NFs and polymeric matrices [119,120,121]. Polymers can undergo various treatments such as acrylation [122,123], mercerization [124], acetylation [125], and treatment with isocyanate [126], maleic anhydride [127,128], water repellents [129], silane [130], peroxides [131], permanganates [132], etc. The most encouraging methodology was the one wherein covalent bond was framed between the fiber and matrix. NFs were amiable to changes due to the hydroxyl functionalities (from cellulose and lignin) that might be engaged with hydrogen bonding within the cellulose particles, subsequently decreasing the action towards the framework [133]. Unlike NFs, the ease of modification of synthetic polymers purely relied on the polymeric structure due to the lack of available functionalities. The major challenges with chemical modifications are the large chemical requirement, chemical waste, difficulties with chemical handling and processing, time and cost of operation, extent of interaction between chemicals and polymers, development of reaction mechanism, and chemical sensitivity of materials [134].

### 2.2. Biological Techniques

Polymeric surface change can be also accomplished via biological methods. Unlike natural polymers, synthetic polymers are somewhat reluctant to undergo biological modifications. Instead of biological modification, researchers performed biological functionalization of synthetic polymeric scaffolds for potential applications [135]. A previous report revealed that the cellulose nanofibrils were kept on the surface of NFs utilizing them as substrates during the maturation cycle of bacterial cellulose [136]. This study showed that around 5–6% bacterial cellulose on the surface of fibers would work on the interfacial bond with polymeric grids. This clever strategy for surface change prompts the advancement of natural and synthetic polymers and different composites with a further developed fiber–network interface. The major difficulties with biological modifications include the liability towards different polymers, time, cost of operation, difficulty with the implementation, sustainability, scalability, area of modification, etc. [137].

### 2.3. Physical Techniques

Various physical processing methodologies were reported for natural and synthetic polymers that resulted in improved mechanical, interfacial, and biological properties depending on their duration as well as intensity [138]. Reported physical modification methods include low- and high-energy radiative methods (X-rays, ionizing radiations, gamma radiations, and e-beams), electrical discharges (thermal or non-thermal plasma, corona discharges, and dielectric barrier discharges), UV and vacuum UV treatments, etc. [139]. These methods resulted in surface-modified fibers and sometimes structurally altered fibers via chemical bond breakage [140,141,142]. Among these, plasma treatment is one of the emerging physical techniques that has been successfully used for surface modification of natural and synthetic polymers, and it helps to improve the mechanical properties significantly [143].

## 3. Plasma Surface Modification (PSM)

Plasma is the fourth state of matter [144]. The phase transitions occur with energy exchanges, and among those, conversion to plasma requires very high energy. Ionizations and bond breakages and formations can occur with high potential. This has led to the development of the “fourth state of matter”, which consists of atoms, ions, molecules, electrons, free radicals, metastable states, etc. [145]. Irving Langmuir termed this unique combination as “plasma” in 1928 [146]. Plasma has additionally been defined by Chen [147] as a ‘quasi-neutral gas of charged and neutral particles which exhibits collective behavior’.

### 3.1. Plasma Modification Strategies

The advances in composite science offer critical freedom for new, further developed, biodegradable and recyclable materials, and these materials can be obtained through PSM. Compared to other treatments, PSM has advantages like lower material consumption, retention of bulk properties, lower processing time, zero waste, and no requirement for further purification or drying of materials after processing and safety. The impact of plasma can be controlled by various parameters such as reactor configuration, gas flow, activity voltage, power, pressure, temperature, and so forth.

Plasma has been classified based on the plasma precursor into three categories [148].

1. Chemically non-reactive plasma: basically, that of monoatomic dormant gases like argon (Ar) which can ionize different atoms or unreactive materials.
2. Chemically reactive plasma: inorganic and natural sub-atomic gases like O_2_, N_2_, and CF_4_, which are highly reactive and never create any polymeric deposition on the substrate surface.
3. Polymer-forming plasma: plasma that is responsive yet shapes a polymeric layer on the substrate (methyl methacrylate (MMA), vinyl pyrrolidone (VP)).

Based on working temperature, plasmas have been categorized as thermal or non-thermal. High pressure and temperature with heavy electrons can lead to thermal plasma generation. Non-thermal plasma or plasma near ambient temperature has been generated under atmospheric pressure or vacuum, as mentioned in Table 3. Thermal plasmas have disadvantages in terms of modification of NFs, most of which are heat-sensitive; therefore, the non-thermal plasma technique has a vital role to play in NF modification [149]. For synthetic polymers, based on their thermal stability, many of them can be easily modified using both thermal and non-thermal plasmas [150,151].

Among all the existing modification strategies for polymers, the better fit is low-temperature/cold plasma (LTP) treatment [153,154,155]. In contrast to other methods, plasma treatment is eco-friendly, has an exceptionally short handling time, is appropriate for thermal-sensitive surfaces, is cost-effective, and provides dry interaction. NFs have low adherence to the matrix due to their smooth surfaces, minimal surface energy, and the absence of functionalities to shape covalent bonds in the fiber–matrix interface [156,157]. PSM can change the morphology of the surface by eliminating the peripheral layer via surface etching, and it influences the surface only to several nanometers in depth [158,159,160]. So, we can conclude that PSM is advantageous over the existing methods. Initially, PSM found application in surface cleaning (O_2_ plasma) [161] and later in surface etching [162], surface functionalization [163], crosslinking [164], etc. An important dynamic species in low-pressure air plasma is oxygen, because it can bring about bonds, abstract electrons, expand oxygen radicals to the polymeric backbone, and is efficient in providing a better NF–polymer interface in composites [165,166,167].

### 3.2. Plasma–Substrate Interaction Mechanisms

#### 3.2.1. Plasma Polymerization

One of the major plasma–substrate interactions is plasma polymerization, which occurs with reactive monomers (mainly containing vinyl bonds) such as acrylic acid (AA) and methacrylic acid. The inelastic collisions occurring in the chamber excite the monomer segments. Furthermore, division and recombination of the same leads to polymerization and in situ deposition on the substrate surface [168]. Plasma polymerization has been targeted to deliver an ultra-thin (10–100 nm), uniform, and stable polymer coating with resistance towards aging, oxidation, contraction, and so forth [169]. Plasma polymers are appropriate for semiconductors and biomaterials. The choice of monomer permits properties of resistance towards erosion, water, chemicals, scratch, grease, heat, penetrability, etc. [169,170,171]. It should be noted that the substrate properties were not unequivocally influenced by this treatment cycle, but the surface morphology was [172].

#### 3.2.2. Plasma-Aided Graft Copolymerization

This could happen either by the generation of dynamic species on the polymeric surface followed by a close approach of the monomer or by the direct joining of monomer and polymer in a plasma environment [173]. In the first case, free radicals are outlined on the polymeric surface through plasma-processing gas [174], but the second case remembers a joined plasma and monomer transparency for one phase using gaseous monomers in the working gas mixture [175]. Both methods are extraordinarily beneficial over traditional ones by offering a huge scope of substance mixtures as monomers, different thicknesses of monomer layers, and restricted annihilation [176,177].

#### 3.2.3. Plasma Ion Implantation

Plasma implantation is another important plasma–substrate interaction mechanism. This process can furnish the substrate surface with functionalities, perhaps the strongest and most sensible modification method. Gaseous particles can usually be activated by plasma, and they can collaborate with the surface. In a brief timeframe, functional groups like hydroxyl, carbonyl, carboxyl, amino, and amido can be deposited on the surface [178]. Likewise, polymer surface properties (hydrophilicity, biocompatibility, mechanical and thermal properties) can be significantly changed through this process [179]. Generally, hydrogen will be distracted first to make radicals at the polymer chain to initiate the mechanism. Other than oxy functionalities, chlorine functionalities such as CCl_4_ can offer improved hydrophilicity [180].

#### 3.2.4. Plasma Ablation

**Figure 2:**
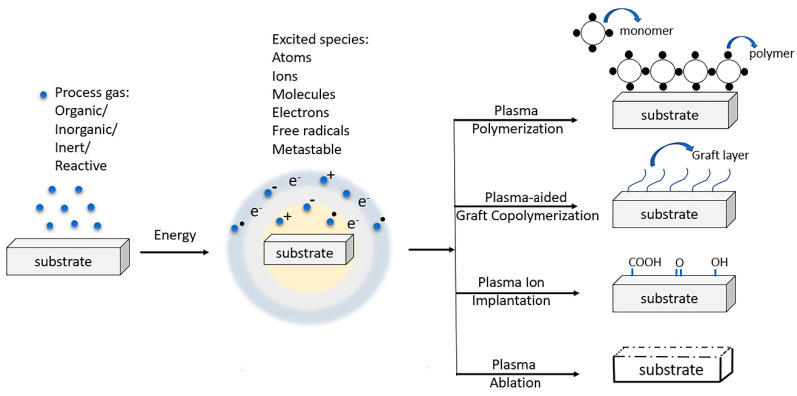
Different interactions between plasma and substrate.

Plasma ablation is the plasma-aided surface etching of materials, very similar to sputtering, without using any liquid etchant. A major share of plasma etching has been carried out with oxygen plasma [181,182,183] where oxygen, the precursor gas, was channeled into a vacuum chamber. During the process, the most peripheral layer of the substrate is etched off, and accordingly, a negligibly small weight loss occurs [184,185]. All the possible plasma–substrate interactions are discussed in Figure 2.

## 4. Applications

During the most recent few years, numerous endeavors have been coordinated using synthetic polymers, NFs, and NFRPCs [138,186]. PSM techniques on polymers have been explored in various applications, such as biomedical and healthcare devices, aerospace, automobiles, environmental pollution remediation, development of biodegradability in existing materials, and so on [187,188]. We attempt to provide comprehensive coverage of potential applications of plasma-surface-modified natural and synthetic polymers in the following sections.

### 4.1. Heavy Metal (HM) Remediation

HMs are a major category of perceived poisons in general prosperity, plants, and animals, considering their harmfulness. For example, HM-contaminated drinking water can cause horrible health issues such as encephalopathy, paleness, nephritic condition, vulnerable muscle coordination, mental growth retardation, etc. [189,190]. HM removal can be accomplished through methodologies like precipitation coagulation [191], membrane separation [192], adsorption [193], electrodialysis [194], and so on. For decentralized water treatment in severely polluted areas and catastrophe zones, adsorption could be pursued. In these areas, it has procured interest as a powerful, adaptable, and cost-effective method for HM removal [195].

Particles having amine [196,197], thiol [198], and -SO_x_ (H) groups (sulfonic acid (SO_3_H) [199] and sulfonate (SO_3_)) [200] as functionalities have been effectively applied for adsorptive removal of various bivalent HMs (Pb^2+^, Cd^2+^, Cu^2+^, Co^2+^, Ni^+2^, and Zn^2+^). In an aqueous medium, material with SO_x_ H groups (possessing negative charge) acts as an ideal electrostatic adsorbent for metallic species [201]. In the past, these functionalities were immobilized onto the particle surface via wet-chemical methods. This could cause further environmental problems in different ways, since it entails a large quantity of chemical usage and disposal. The substrates need not fulfill any criteria to be used for PSM, i.e., they can be of any size, shape, or morphology, and no surface activation is required in most cases. Plasma treatment parameters could be changed accordingly to achieve homogenous modification [202,203]. The list of the most dangerous HM pollutants includes but is not limited to arsenic (As), lead (Pb), mercury (Hg), cadmium (Cd), chromium (Cr), copper (Cu), zinc (Zn), and so forth [204].

**Figure 3:**
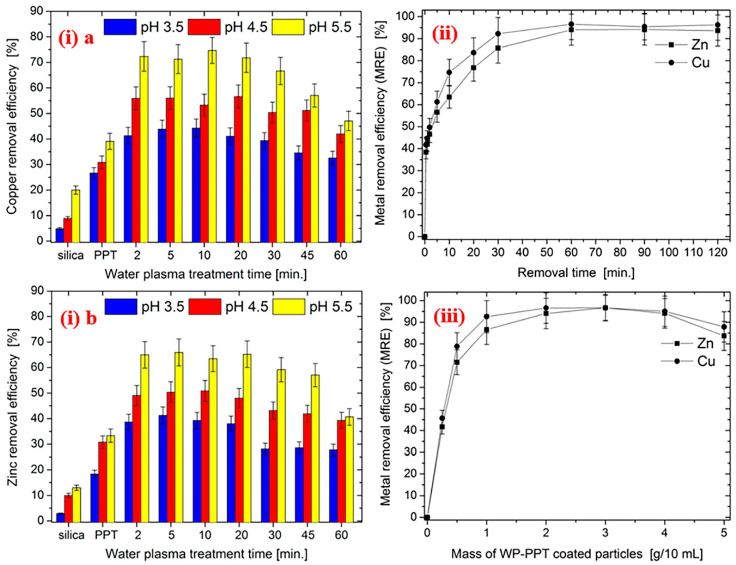
MRE for WP-PPT-coated particles as a function of (**i**) plasma treatment time and pH of solution for (**a**) Cu and (**b**) Zn, (**ii**) time of contact, (**iii**) mass of adsorbent [205], with permission from ACS Publications, 2015.

An effective adsorbent for HM can be manufactured by plasma polymerization, which can outfit active functionalities for HMs. Akhavan et al. [205] reported the plasma-assisted fabrication of negatively charged particles on silica for HM removal. Sulfur-rich groups were deposited with thiophene plasma, which further converted into sulfonic acid and sulfonate with water plasma. Figure 3 illustrates the impact of various parameters (pH of the medium, duration of water plasma, duration of adsorption, and mass of adsorbent) on metal removal efficiency (MRE). Both pristine and water-plasma-treated-plasma-polymerized-thiophene (WP-PPT)-coated particles show altogether higher MREs for all attempted pH values. Increased MRE with duration of plasma was intended for both Cu and Zn particles, but with time, it diminished.

Pb and Hg are profoundly harmful metals, and their wide use has led to broad environmental and health issues. There are diverse pathways for these metals to contaminate the environment, such as fertilizers and pesticides, automobile exhaust, battery industries, metal plating and finishing, melting, and smelting of ores, and additives in gasoline and pigments. One of the major sources of Pb and Hg contamination is petrochemical ventures. Plasma-assisted surface tailoring of silane substrates for the removal of Pb and Hg was successfully established by Boroujeni et al. [206]. Polyether sulfone (PES) membranes were functionalized with thiol groups via plasma treatment to improve the membrane selectivity as well as wettability during the filtration of HM solution. PES membranes with nanoporous structures were made by the phase inversion technique. Ar/O_2_-plasma-pretreated PES was immersed in the dispersion of 3-mercaptopropyltrimethoxysilane in ethanol. The results revealed the improved adsorption efficiency of membranes with thiol-functionalized nanosilica towards Hg and Pb ions. The polar groups like −OH, −OOH, or −CO on the PES surface offered improved hydrophilicity as demonstrated by Steen et al. [207]. The thiol (−SH) functionalization advances HM adsorption by chelation with Hg^2+^ and Pb^2+^ [208]. Removal of Cd^2+^ is challenging compared to Pb^2+^ due to the poor Cd^2+^-binding capacity of thiol-functionalized silica, as demonstrated by Liang et al. [209].

**Figure 4:**
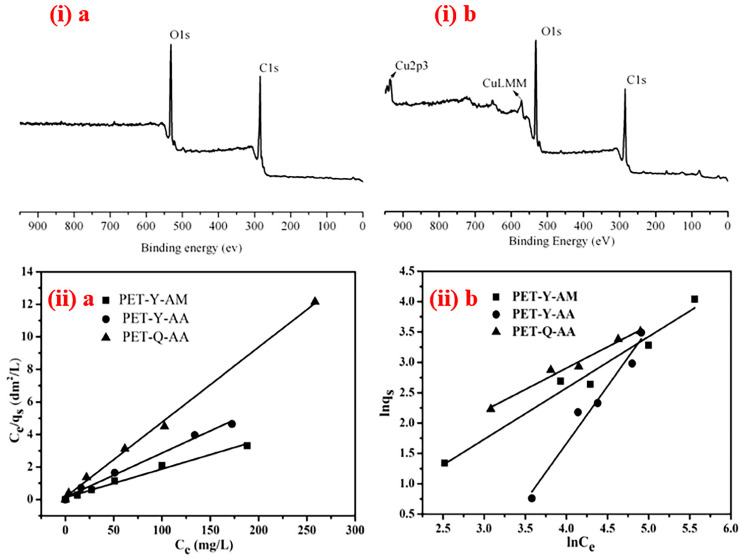
PET-AA nanofibrous membrane (**i**) XPS spectra a. before and b. after Cu^2+^ adsorption, (**ii**) adsorption isotherms according to (**a**) Langmuir equation and (**b**) Freundlich equation [210], with permission from Elsevier, 2010.

Furthermore, plasma surface engineering has been extended to polymers and NFs for HM removal. Ding et al. [210] successfully used AA and acrylamide (AAm)-grafted polyethylene terephthalate (PET) nanofibrous films to remove Cu^2+^ from solution. PET nanofibrous membranes furnished with carboxyl and acryl amino groups have high reusability and could provide efficient sorption of Cu^2+^. The detailed characterizations revealed the geometrically uniform nanofibrous defect-free structure of the membrane, successful grafting of carboxyl and acryl amino groups, improved hydrophilicity, and the presence of Cu^2+^ adsorbed on the surface of the modified membranes. The electrospun (ES) layers before and after Cu^2+^ adsorption was examined by XPS analysis, and the kinetics of adsorption by Langmuir and Freundlich isotherms (Figure 4) successfully revealed the efficiency of PSM on the MRE.

**Figure 5:**
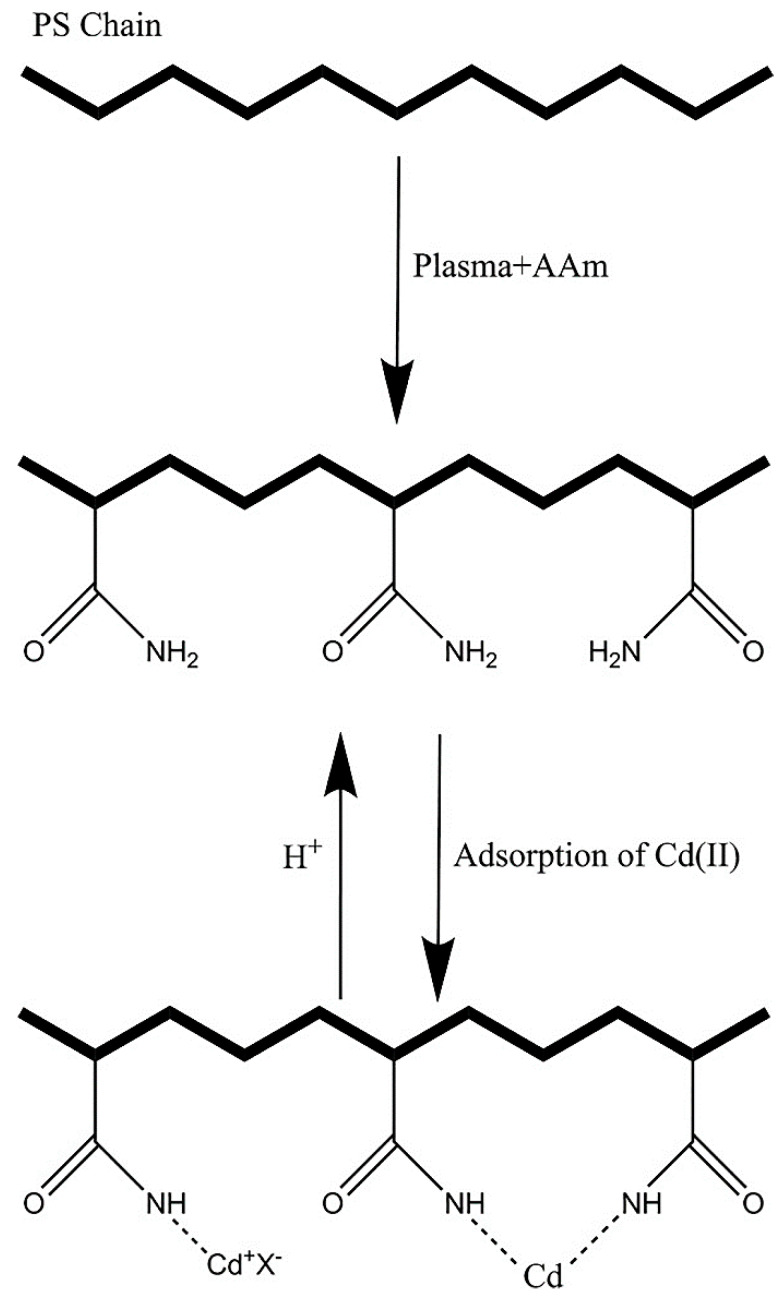
A schematic representation of nanoadsorbent preparation via AAm plasma and HM adsorption [211], With permission from Elsevier, 2016.

N_2_ plasma is an active tool for the surface functionalization of polystyrene (PS) with amide (-NCO-) and amine (-NH-) groups [211]. This work targeted the development of AAm-functionalized ES PS nanofibrous samples as a promising nano adsorbent. According to Chan et al. [212], NH_3_ plasma was more capable of creating −NH_2_ groups on a polymer surface than N_2_ plasma because of its inherent properties. NH_3_ plasma usually creates higher amounts of radicals. Simultaneously, many radicals are converted into certain inappropriate functional groups, but N_2_ plasma cannot. From the atomic absorption spectroscopic (AAS) analysis, the high adsorption of ions occurred at pH 5 in the order of Cd^2+^ (10 mg/g) and Ni^2+^ (4.9 mg/g) by using the adsorbent dosage of 1 g/L. Surface functionalization with AAm monomer and the Cd^2+^ adsorption by ES PS nanofibers is schematically exhibited in Figure 5.

PP non-woven fabric has been widely used as an adsorbent due to its higher flexibility and chemical stability compared to other polymers [213]. Surface-tailored PP with reactive carboxylic functionalities via AA plasma, grafted with chitosan, was used to capture HMs. Chitosan was up to three times more efficient than chitin towards HMs, which proves the importance of the amino groups [214]. AA was loaded onto PP fibers through LTP followed by gaseous phase grafting. This was further amidated with triethylenetetramine and subjected to imprinting with a Cr (VI) template. The modified polymer exhibits better hydrophilicity and selective Cr (VI) adsorption.

Instead of chitosan, researchers attempted cysteine for HM remediation. Vandenbossche et al. [215] reported that a cysteine-immobilized non-woven PP geotextile was an innovative and eco-friendly HM adsorbent. Reactive carboxylic functionalities were implanted on the PP surface by AA plasma followed by cysteine grafting. The sorption of HMs such as Cd (II), Pb (II), Ni (II), and Cu (II) was reported by using L-cysteine and poly-L-cysteine [216,217]. Another work based on PP was reported by Ozmen et al. [218] on a novel adsorbent for HMs (Cu (II), Co (II), Cr (III), Cd (II), and Pb (II)) using PE-coated PP fiber by plasma-polymerized deposition of glycidyl methacrylate. This was further functionalized with 4- amino benzoic acid. Later, Nia et al. [219] developed AA-plasma-treated PP as an adsorbent for cesium (Cs), which is commonly emitted during nuclear accidents. The specific adsorption capacity towards Cs was shown by Prussian blue. O_2_-plasma-treated PP was used as the supporting material to immobilize Prussian blue. PP was surface tailored with carboxylic acid groups via polyacrylic acid in the O_2_ plasma environment.

Peng Wei et al. [220] reported a PP non-woven fiber adsorbent for HMs with an improved surface hydrophilicity. Higher MRE was achieved by grafting chitosan and 1- vinyl imidazole onto the PP non-woven fiber. Surface roughness, hydrophilicity, and surface functionalization were achieved through LTP processing. The adsorption efficiency of the reported adsorbent was positively correlated with time and concentration. A total of 1 g of fabric could trap 28.21 mg Cu ions with an adsorption duration of 300 min from 1.0 g/L Cu solution. The adsorption efficiencies of reported HM adsorbents which were developed/modified with LTP treatment are discussed in Table 4.

### 4.2. Biomedical Applications

The PSM of polymers has been used for many potential applications in biomedical science and engineering because of its ability to enhance protein absorption [230], immobilize biomolecules, induce stable superhydrophobic and superoleophobic behavior [231,232,233], and its biocompatibility and self-cleaning properties on the surface [234]. Superhydrophobic and super hydrophilic surfaces could be fabricated with various plasmas such as poly methyl methacrylate (PMMA) [235], polycaprolactone (PCL) [236], polyether ether ketone (PEEK) [237], and so on. Polymer surfaces can be decorated with oxy and nitro functionalities with LTP treatment using O_2_, air, N_2_, or NH_3_ [238,239] as precursors. These polar hydrophilic groups are formed by the interaction of chemically active species generated by gas discharge plasma with different groups in the polymer molecules. In contrast to O_2_ or N_2_ plasmas, helium (He) and Ar discharges lead to free radicals [240,241], which can subsequently be used for crosslinking or grafting oxy functionalities with exposure to air or O_2_ plasma [242].

Biodegradable aliphatic polyesters have been recognized broadly as a platform material for tissue engineering [243] due to their mechanical properties, low immunogenicity, and biocompatibility [244]. However, the low surface energy of these polymers leads to poor cell adhesion and proliferation [245]. It was reported that various protein components for the extracellular matrix such as gelatin, laminin, fibronectin, and collagen could be immobilized on the polymer surface by plasma processing to improve the cell proliferation rate [246,247]. - PSM has become a better candidate in tissue engineering because of its ability to improve biocompatibility [248] and to modify the inner surface of porous objects [249]. Polymers combined with nanoparticles of bioactive glass for orthopedic applications have been reported by Leite et al. [250]. A functional substrate such as micro-arrays, cell arrays, biocompatible surfaces for medical applications, microfluidics for diagnosis, and many more [251] can be made by immobilizing biomolecules on a substrate surface. Later, the influence of silver-doped bioactive glass on the antibacterial bio-adhesive layer was also confirmed [252], which found application in orthopedic surgery. The durable bioactivity in solution as well as in freeze-dried storage was correlated with the hydrophilic nature of the treated surfaces [253].

**Figure 6:**
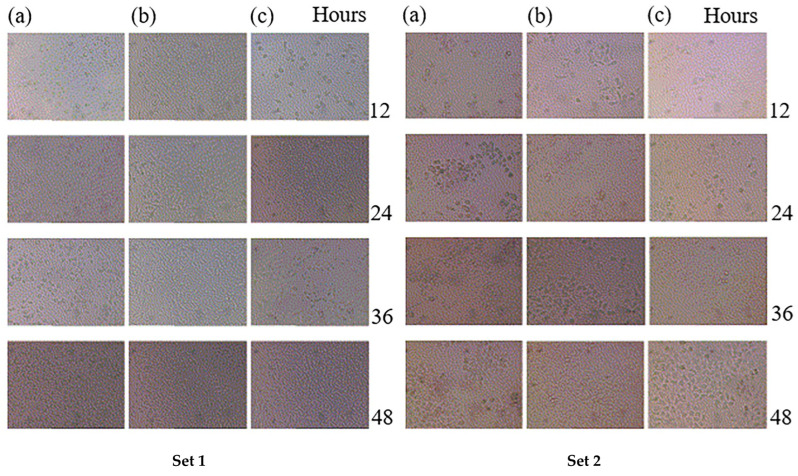
Microscopy photographs of cells after 12, 24, 36, and 48 h of plating. Set 1, L929 (**a**) chitosan grafted PLLA, (**b**) pristine PLLA, and (**c**) glass control. Set 2, L02 (**a**) chitosan grafted PLLA, (**b**) pristine PLLA, and (**c**) glass control [242], with permission from Elsevier, 2004.

A few more endeavors have been made regarding the PSM of PLA. Ho et al. [254] found an improved cell affinity by plasma-aided surface immobilization of RGDS (Arg-Gly-Asp-Ser) on poly-l-lactic acid (PLLA) scaffolds. Surface functionalization of biodegradable PLLA was achieved by plasma coupling reaction of chitosan [242]. L929 (mouse fibroblasts) and L02 (human hepatocytes) cell lines cultured on the modified scaffold have shown spreading. The cells grown on the surface proliferated at almost the same rate as those cultured on glass (Figure 6).

PP is one of the most reluctant polymers towards modifications due to its high chemical barrier responses and structure. Recent reports indicated that plasma-surface-engineered PP with various optimized process variables [255] has improved surface hydrophilicity and adhesive properties of PP or poly(ethylebe terephthalate) (PET) [256,257]. Grenadyorov et al. reported that the plasma-assisted deposition of silicon and oxy functionalities incorporated with hydrogenated amorphous carbon films onto PP improved the antithrombogenicity and hemocompatibility [258]. Blood compatibility with the deposited film was evaluated by an in vitro investigation of platelet adhesion and cytotoxicity.

Lukowiak et al. studied the ability of polyglycerol plasma to improve the protein and bacteria resistance of the PP surface [259]. They performed PSM to anchor Br on the PP surface, which was further replaced by amine groups from hyper-branched polyglycerol. Later, Tsou et al. [260] used the immersion-pad-pressing-drying-plasma (IPDP) process to modify the non-woven PP inert surface by grafting methyl diallyl ammonium salt. They used O_2_ and Ar as the purge gases, and the grafted PP surface showed excellent antibacterial as well as hydrophilic properties. The use of Cu and Ag was already reported regarding the antibacterial property of materials [261]. Woskowicz et al. [262] investigated the advantage of plasma-aided surface tailoring of PP with Cu or Ag over the sputter coating.

The ability of Ar plasma to alter surface morphology, wettability, and biocompatibility was already reported by Gomathi et al. [263]. Later, Ar plasma was used to modify the PP surface to create a nano-embossed structure for improved surface adhesion, wettability, and biocompatibility. With a longer treatment time, the 2D surface of PP changed to 3D with a long nanofiber-like structure. Further characterization revealed the crosslinking of polymer chains and an increased amount of conducting amorphous carbon on the surface. Hana et al. [264] reported the use of atmospheric pressure plasma to make the PP surface biocompatible. A nanolayer polymer was deposited on PP using plasma of a mixture of propane and butane in N_2_. The thin layer was highly wetting, smooth, flexible, homogeneous, and had good adhesion to the PP surface; hence, it was a better candidate for technical and biological applications.

The bone regeneration capability of recombinant human-bone-morphogenetic-protein-2 (rhBMP-2)-immobilized Medpor using AA plasma polymerization was studied by Lim et al. [265]. Plasma treatment improved the surface wettability by tailoring the surface with carboxyl groups which improved the protein immobilization. The activity of the Medpor surface towards cells demonstrated the potential of PSM to immobilize the protein on a polymer-based implant, which could be vital in bone tissue engineering.

In the past decades, the number of hernia mesh surgical implantations increased enormously. The commercial hernia mesh was composed of PP due to its biocompatibility, physical properties, inertness, ease of processing, and moldability. However, there were challenges, such as diminished long-term strength, high adhesion to the abdominal wall, lack of biocompatibility, a hydrophobic surface that resisted drug adhesion, and foreign body rejection. Saitaer et al. [266] developed a polydopamine-coated mesh for hernia surgery with improved surface wettability. The PP mesh surface was activated with O_2_ plasma and further coated with polydopamine. Thereby, the adhesion and release of the drug, levofloxacin, became easy. The drug-loaded mesh has shown improved antimicrobial properties, and the drug release lasted for at least 24 h. Houshyar et al. [267] successfully incorporated nanodiamond with PP (ND-PP) for making hernia mesh with improved long-term stability, mechanical strength, and biological performance. The ND-PP mesh surface activation was done with oxygen plasma.

The use of plasma-aided graft polymerization of (vinyl benzyl) trimethylammonium chloride (VBTAC) on the surface of PE followed by plasma surface activation with air was reported by Kliewer et al. [268]. VBTAC is a contact-active antibacterial quaternary ammonium salt. The poly-VBTAC-modified PE had a homogeneous layer with a thickness of 300 nm and high charge density. The antibacterial evaluation against Gram-positive (*S. aureus*, *S. epidermis*) and Gram-negative (*P. aeruginosa*, *E. coli*) bacteria revealed the efficiency by eradiating all inoculated bacteria. The contact activity was confirmed by an agar diffusion assay. Therefore, this can be used for packaging applications and green antimicrobial management without biocide release. Antibacterial PE surfaces were developed by Popelka et al. [269] using LTP treatment with substances containing antibacterial groups such as triclosan (5-Chloro-2-(2,4-dichloro phenoxy) phenol) and a chlorhexidine (1,1′-Hexamethylenebis [5-(4-chlorophenyl) biguanide]).

**Figure 7:**
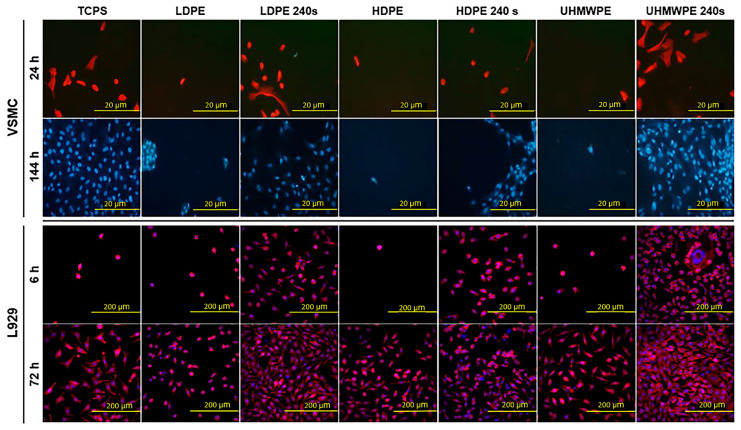
VSMCs and L929 cells adhered (6 or 24 h) and proliferated (72 or 144 h) on pristine and plasma-treated PE (LDPE, HDPE, UHMWPE) (Reproduced from Reznickova et al., Mater. Sci. Eng. C 2015, 52, 259–26 [270], with permission from Elsevier, 2015.

Tissue engineering has shown vital growth in the past two decades and trended towards the application of polymer-based implants. Plasma surface activation and property dependents of biomedical polymers (low-density polyethylene (LDPE), high-density polyethylene (HDPE), and ultra-high-molecular-weight polyethylene (UHMWPE)) were examined by Reznickova et al. [270]. The surface activation was performed with Ar plasma, and the cytocompatibility was examined using vascular smooth muscle cells (VSMCs) as a model for vascular graft testing and mouse fibroblasts as connective tissue cells (L929). PSM shows improved cell adhesion and proliferation, as shown in Figure 7. Pandiyaraj et al. [271] developed an antifouling LDPE surface using atmospheric-pressure-LTP-assisted copolymerization of an AA and polyethylene glycol (PEG) mixture. Further improvement in various properties was achieved by functionalizing the modified PE with chitosan. Protein adsorption, platelet adhesion, and final in vitro studies revealed the improved antifouling nature of the modified matrix. The reported works of plasma-treated polymers for biomedical applications were summarized in the Table 5.

### 4.3. Water Purification

Water and energy deficiencies are worldwide concerns of developing seriousness. Around the world, over a billion groups seek access to clean drinking water, a central human need. Quick industrialization has posed a huge danger to human well-being and climate due to the expanded release of wastewater, primarily containing inorganic and natural toxins. Wastewater release can create an engaging scope of contamination, which will not only expand the chemical oxygen demand (COD) and harmfulness of sewage, but also reduce the level of light infiltration and repress the photosynthesis of aquatic plants along these lines, causing the annihilation of the amphibian framework [272,273]. Reverse osmosis (RO) is one of the major technologies to produce fresh water from saline and other wastewater sources.

Polysaccharide-based adsorbents such as cellulose and chitosan are efficient adsorbents towards organic and inorganic pollutants from water, even in their raw form [274]. The cost-effectiveness, poly functionalities, abundance, specific entrapment of pollutants, diverse architecture, and reactivities of lignocellulosic materials identified them as the perfect candidate for adsorptive removal of pollutants from water [275].

**Figure 8:**
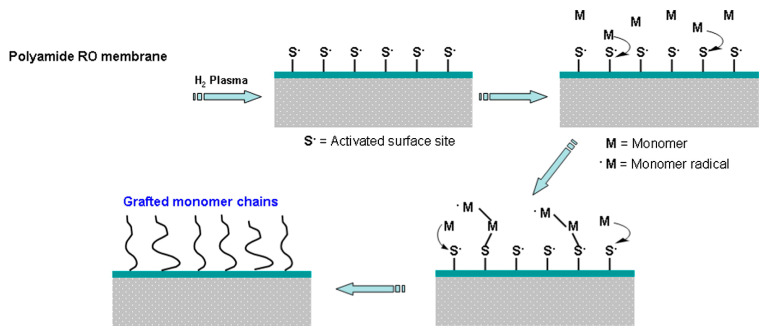
Polyamide RO membrane modification via plasma-induced surface activation followed by surface graft polymerization [283], with permission from Elsevier, 2012.

Another significant method for water purification is RO, but membrane fouling was the major challenge with this. The thin film composite RO membranes have been effectively modified with O_2_ and Ar plasma [276], and PP microporous membranes with N_2_ plasma [277], CO_2_ plasma [278], and air plasma [279], to improve the antifouling property. Zou et al. [280] grafted trimethylene glycol dimethyl ether hydrophilic polymer on aromatic polyamide RO membrane to reduce the organic fouling tendency. Antifouling properties and improved flux performance for RO membranes were successfully developed through surface nano structuring aided with atmospheric-pressure-plasma-induced graft polymerization [281,282] (Figure 8). The membrane surface was activated with atmospheric plasma followed by solution-free radical graft polymerization of water-soluble monomers such as methacrylic acid and AAm. The resulting membrane offers lower mineral-scaling propensity and high permeability as compared to the commercial membrane.

Chitosan is a linear polysaccharide obtained by the deacetylation of chitin. Chitosan has drawn extraordinary consideration as a biopolymer in the field of dye toxin evacuation, even at low concentrations (ppm or ppb levels). It has many benefits, such as inexhaustible nature, eco-friendliness, cost-effectiveness, biocompatibility, biodegradability, and abundance [284,285]. Chitosan contains an enormous number of dynamic hydroxyl and amino groups, which offers a high level of entanglement towards natural dyes and inorganic HMs. Wen et al. [286] designed another methodology for modifying chitosan by utilizing glow discharge plasma to discover applications as adsorbents for dye removal from aqueous media. The morphology and crystallinity of chitosan particles were changed, and the number of methyl groups was increased by the plasma treatment. The dye adsorption tests revealed the quicker capture productivity of plasma-modified chitosan for dye expulsion compared to untreated chitosan.

Gopakumar et al. effectively used plasma-modified polyvinylidene fluoride (PVDF) for the adsorptive removal of toxic dyes and metallic nanoparticles from water [287]. ES PVDF was modified with CO_2_ plasma to achieve hydrophobicity. The surface decoration with −COO groups was used for removing crystal violet dye and iron oxide (Fe_2_O_3_) nanoparticles from water. Instead of CO_2_, ES PVDF modifications were attempted with CF_4_ for air gap membrane distillation (AGMD) [288]. In that case, newly formed CF_2_-CF_2_ and CF_3_ bonds brought down the surface energy of the layer and imparted omni-phobic properties. This prevented the film from wetting with low-surface-pressure fluids such as methanol, ethylene glycol, and mineral oils. Jie et al. [289] developed a poly (AAm-co-AA) hydrogel using glow-discharge-plasma-induced copolymerization for adsorptive removal of toxic dyes such as crystal violet and methylene blue. Plasma-modified natural shells (almond shells) for adsorptive removal of Eriochrome-Black-T [290] and Jatropha curcas shell for reactive red 120 textile dye [291] were reported. Since water bodies were contaminated with various pollutants, Takam et al. [292] developed a multi-component adsorption system out of cocoa shell which has an optimal capability towards Azur II (cationic dye) and reactive red (anionic dye). The amount of hemicellulose and lignin was reduced by plasma treatment, and the shell became more hydrophilic and an efficient adsorbent. The reported works of plasma- treated polymers and their derivatives for water purification were summarized in the Table 6. 

### 4.4. Packaging Applications

The major share of packaging materials is covered by paper, plastic, glass, and metal in the packaging industry. Plastic plays a vital role due to its flexibility, cost-effectiveness, low weight, heat-sealing capability, durability, and satisfactory mechanical strength [293,294,295]. The use of synthetic polymers in various aspects of day-to-day life has been questioned because of their non-degradable nature and other possible harms [296]. Researchers developed edible biodegradable polymer films derived from polysaccharides, proteins, and lipids [297,298]. The technological applications were hindered by their inherent disadvantages, such as poor mechanical properties, wettability, printability, and adhesive nature [299,300]. Many of the reported works revealed the efficiency of LTP treatment for altering mechanical properties, water vapor permeability, antimicrobial properties, etc. [301,302,303].

For packaging applications, the materials should have good surface and barrier properties. Arolkar et al. [304] reported the use of air-plasma-treated corn starch/poly(ε-caprolactone) (CSPCL) films for packaging applications. The biodegradation and surface properties with plasma treatment at various time intervals revealed the increased surface roughness and adhesive properties. The impact of plasma treatment on the biodegradation of control and treated films was analyzed by simulating natural conditions in a controlled environment using an indoor soil burial method with a system containing Bacillus subtilis, a commonly occurring soil bacterium. The hydrophobic thermoplastic film made of corn starch was invented by Daniele et al. [305] with SF_6_ plasma to improve hydrophilicity. Later Anastácia et al. [306] analyzed the corn starch film modified using SF_6_ plasma with a capacitively coupled plasma-enhanced chemical vapor deposition (CVD) reactor. Instead of SF_6_ plasma, Sifuentes-Nieves et al. [307] used hexamethyldisiloxane (HDMSO) plasma and achieved improved barrier performance against water. Later, they analyzed the effectiveness of HDMSO plasma on three different granular corn starches (normal, Hylon V, and Hylon VII) [308].

Many trials have been conducted with chitosan, a biodegradable polymer, to achieve promising surface properties for application in the packaging industry. Assis et al. [309] investigated the water vapor, CO_2_, and O_2_ permeability of a chitosan surface with LTP-deposited hexamethyldisilazane. Later, Chamchoi et al. [310] modified the chitosan with Ar plasma, and Chen et al. [311] attempted a film composite of zein and chitosan, which was further modified with LTP treatment. The treated composite film exhibited optimum tensile strength, enhanced water vapor barrier, and improved thermal stability. The improved properties were due to the ordered secondary structure of zein molecules and the occurrence of more H-bonds between zein and chitosan with PSM.

One of the major challenges in the food packaging industry has been the development of a gentle process concept that can keep food natural, minimally processed, and safe. Ulbin-Figlewicz et al. [312] developed an antibacterial edible chitosan film by low-pressure plasma treatment. They determined the antibacterial activity of chitosan film incorporated with lysosomes exposed to He plasma. The edible film was prepared using low-molecular-weight chitosan by casting from a lactic acid solution with a solution of the lysosome. The dried films were further modified using He plasma and tested against the growth of listeria monocytogenes, yersinia enterocolitica, and Pseudomonas fluorescens. Instead of chitosan, Helena et al. [299] used cassava starch for packaging film. To avoid the expected difficulties due to the hydrophilicity of starch, they developed a multi-layer film of PLA-starch and PCL-starch. The capability of air plasma to improve the surface roughness was utilized in this case, and thereby interlayer adhesion was ensured. Song et al. [313] reported O_2_-plasma-treated PLA as a packaging material without any other coating materials.

The safety aspects of LTP technology are very important, and limited studies have focused on the formation of toxic components due to the interaction of plasma-induced species, polymers, and various foods [314,315]. A significant increase in the peroxide content in nuts with plasma treatment was reported by Thirumdas et al. [316]. The possibility of undesirable derivatives such as aldehydes, keto acids, hydroxyl acids, and short-chain fatty acids was also discussed. All the studies mentioned here finally emphasized the necessity of safety and risk assessment of the effect of PSM on various polymers and food products. The reported works on the PSM of NFs, polymers, and their composites were summarized in Table 7.

### 4.5. Sensor Development

Polymeric sensors have received attention in monitoring the human-inhabited environment. Recent advancements in polymer-based touch sensors have been utilized in robotic medical procedures such as prosthetics, surgery, catheter radiofrequency ablation, and artificial skin development [206]. Here we attempted to summarize the major advancements achieved in the use of plasma technology to develop polymer-based sensors.

**Figure 9:**
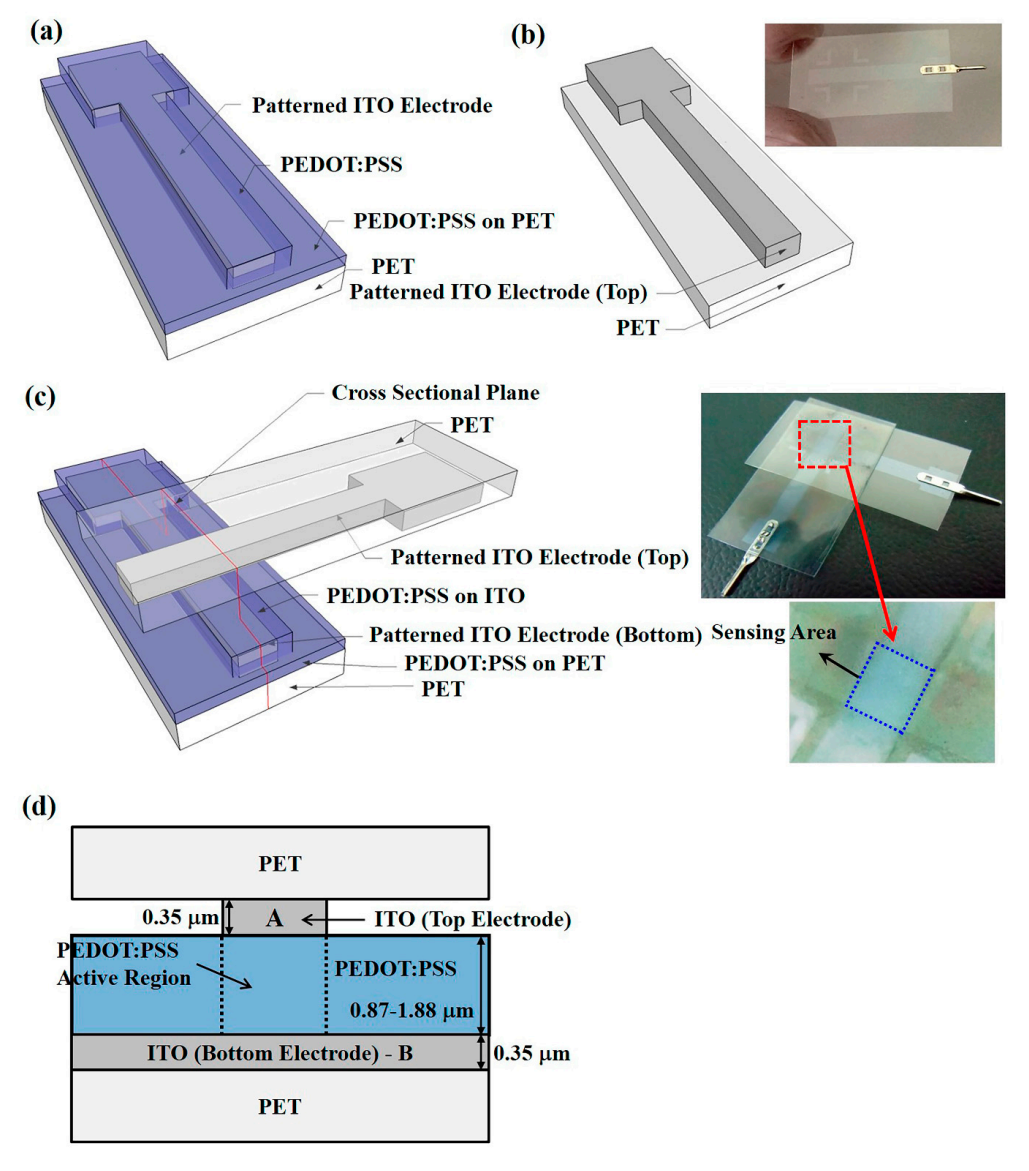
Schematic representation of PEDOT: PSS pressure sensor (**a**) part I, (**b**) part II, (**c**) final device, and (**d**) cross-sectional diagram of the sensor [328] under Creative Commons license.

Lee et al. [327] reported a stretchable and transparent touch sensor using selective plasma-based patterning. Thin PU dielectric film was sandwiched between electrode lines made of silver nanowires and graphene oxide on polydimethylsiloxane (PDMS). The hydrophilicity of PDMS was improved using O_2_ plasma. Another highly efficient pressure sensor was reported by Wang et al. [328] by combining an Indium-Tin-Oxide (ITO) electrode with plasma-modified (3,4-ethylene dioxythiophene) and polystyrene sulfonate (PSS), as shown in Figure 9. The piezo-resistive sensitivity and response were enhanced by N_2_ plasma. The advantages of a polymer-based pressure sensor were the lowest wear resistance and ease of surface modification.

Like pressure, temperature and humidity are vital parameters in health and the environment [329,330,331]. Aliane et al. [332] developed a high-accuracy and stable temperature sensor printed on a flexible foil made of polyethylene naphthalate (PEN) and PET. The temperature-coefficient-sensitive layers were made using antimony tin oxide (ATO), where the resistance temperature coefficient was improved by O_2_ plasma, because even if the O_2_ plasma was highly aggressive, short-duration plasma treatment can enhance the temperature sensitivity by providing surface roughness and surface activation towards the ATO layer on the surface of PET and PEN polymer layers.

**Figure 10:**
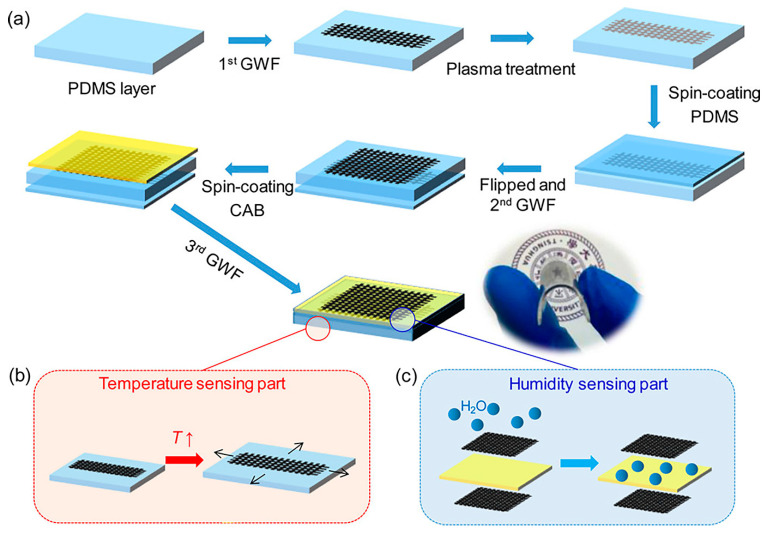
(**a**) Schematic diagram of the path to final FTHS. (**b**) Mechanism for temperature sensing. (**c**) The mechanism for humidity sensing [334], with permission from ACS Publications, 2017.

Spin-coated PMMA on a chromium IDC structure on a glass substrate for humidity sensing was reported by Dhabade et al. [333] As per the literature, this was the first reported work on the use of a plasma-modified polymeric material for humidity sensing. In this work, Ar-plasma-treated PMMA was used as a dielectric component for relative humidity sensing. Later, Zao et al. [334] developed an efficient sensor for both temperature and humidity by sandwiching an O_2_-plasma-treated graphene-woven fabric (GWF) sensor between PDMS layers. The spin-coated layer of cellulose acetate butyrate on the GWF film helped to achieve humidity sensing capability. In this flexible sensor, the temperature- and humidity-sensing compartments were stacked in a layer, as shown in Figure 10, which offers high sensitivity and negligible mutual interference.

Another important category was gas sensors because a significant amount of volatile organic chemical (VOC) pollutants is released into the atmosphere from chemical treatment plants and automobile exhaust. Polymeric gas sensors mostly work with an electrical response method and can be used for the detection of individual vapors as well as complex gases [335,336,337]. Tang et al. [338] developed a nanowire chemiresistive gas sensor by nanoscale soft lithography specifically for NH_3_ and NO_2_ with PEDOT: PSS using O_2_ plasma. With the exposure of nanowire (p-type) to NH_3_, molecules interacted through electron transfer and were adsorbed. The acceptance of electrons from NH_3_ led to the loss of charge carriers. Conversely, the desorption induced the electron consumption in the chain which led to increased conductivity of the sensor recovering towards its initial value. Recent advancements in this field extended to plasma-modified complex polymer composites for gas sensors. Rivera et al. [339] tuned the chemiresistive properties of polyaniline (PAni)/multiwalled carbon nanotube (MWCNT) composite doped with l-carrageenan with Ar plasma to improve the conductive sensing ability towards H_2_.

Gradually, polymer-based sensor research was extended to biomedical applications. Organic semiconductive polymers were explored more for the detection of simple electrolytic salts, enzymes, neurotransmitters, etc. [340]. Organic electrochemical transistors have been used for enzymatic sensing through dedoping or field effect transistor principles which have been combined with PSM to obtain flexible and optically transparent biosensors [341,342]. Based on the cathodic detection of O_2_ consumed by glucose oxidase reaction, Maekawa et al. [343] developed a sensor for glucose detection by immobilizing the enzyme on O_2_-plasma-treated PDMS. O_2_ plasma was utilized to replace silane groups with silanol groups. One of the recent developments in polymer-based sensors was extended to the transformation of LDPE aided with plasma treatment to an electroactive material capable of sensing dopamine and glucose [344,345]. Dopamine is an important neurotransmitter in disorders like Parkinson’s, schizophrenia, etc. The functionalities formed by plasma treatment led to the oxidation of dopamine, producing dopamine-o-quinone. The sensing capability has been further augmented by incorporating gold with a polymer surface [346,347]. Table 8 summarized the reported studies of plasma-treated NFs, polymers, and their derivatives for sensing applications.

## 5. Plasma-Modified Natural and Synthetic Polymers—Future Perspectives

PSM has been widely explored so far, but there is still much work to be done. The plasma technique contributes significantly to the economic prosperity of industrialized societies. The emerging application of plasma includes optics, glass, energy, aerospace, automotive, plastics, textiles, medicine, hygiene, and the environment. The increased demands for optical elements compatible with intense lasers and other radiation technologies illuminate the use of plasma devices as filters and mirrors for which conventional methods were unable to provide satisfactorily. PSM is a versatile and compatible modification methodology for polymers to achieve abrasion resistance, chemical stability, antifouling properties, and biocompatibility for biomedical applications. An article entitled ‘white paper on the future of plasma science and technology in plastics and textiles’ [352] highlights the existing efforts and frontiers of plasmas in detail. The therapeutic effects of LTP processing were expected in biomedical fields, including the prevention of organ adhesion, cell proliferation, hemostasis, and vascularization. Researchers started using non-thermal plasma in cancer treatments because of existing challenges, such as the existence of reactive species scavengers and the shallow infiltration of plasma on the tumor surface. LTP processing is an emerging anticancer technology that can generate unique reactive oxygen and nitrogen species for cancerous cell elimination. However, the effect of physical interactions between cells and the extracellular matrix in the tumor microenvironment on the plasma therapy outcome is unknown [353,354]. The current scenario predicts an upsurge in the demand for NFs and their composites. The major limiting factors to the demand of NF-based sustainable materials, such as moisture absorption, lack of wettability, and poor compatibility with the polymer matrix, can be successfully addressed by PSM. 

The community of plasma scientists strongly believes that more exciting advances will continue to foster innovations and discoveries in the next decades. The future directions of research will be significantly connected with two terms: machine learning (ML) and artificial intelligence (AI). These most efficient, digital, and high throughput methods can be used to identify and create next-generation advanced materials and designs and to evaluate their feasibility in various applications. AI and ML can offer a virtual base in material development via material informatics, computational modeling, and simulation studies [355,356,357]. AI-incorporated plasma processing has already started in neuromorphic engineering [358]. Since plasma is an exciting system of particles, it can offer unpredictable reactions with various parameters. ML has started focusing on modeling, diagnosis, and control of equilibrium plasmas [359]. The current and future trend in this field points towards emerging applications of ML/AI in plasma engineering for recycling of plastics, pollution remediation, water purification and in circular economy for sustainability. in.

## 6. Conclusions

Investigation attempts in the field of potential applications of plasma-surface-modified NFs and polymers have shown tremendous improvements in the last two decades. In this review, the extraordinarily unique features of NFs, polymers, their derivatives, and various modification methodologies, especially PSM, for various applications have been established. The potential applications include HM sensing, wastewater treatments, biomedical applications, packaging applications, various sensing applications, etc. PSM played a vital role in surface activation, surface functionalization, and changing surface morphology. In this way, surface properties such as hydrophilicity, adhesion, and surface energy can be altered. There was a surge in the use of NFs in the last decades in composite applications due to their surface affinity amelioration towards the matrix after plasma treatment. Likewise, an increased demand of plasma-modified NFs was observed in various applications. PSM has a large role to play in the coming decades with computational tools like AI and ML. Modeling and simulation, real-time monitoring, and control of non-equilibrium plasma can be potentially transformed with these emerging techniques. The field of plasma-modified NFs and their derivatives is seemingly ripe for application-driven advancement in the future. The current article attempts to give an overview of the reported works in this field so far and hence will have a remarkable impact on future research on plasma driven surface engineering of sustainable polymers for circular economy.

## Figures and Tables

**Table 1:** Commercially significant fiber sources and gross production across the world [36], with permission from Elsevier, 2012.

Fiber Sources	Production across the World (10^3^ tons)
Sugarcane	75,000.00
Bamboo	30,000.00
Jute	2300.00
Kenaf	970.00
Flax	830.00
Grass	700.00
Sisal	378.00
Hemp	214.00
Coir	100.00
Ramie	100.00
Abaca	70.00

**Table 2:** Reported some works on NF composites.

Sl. No:	NFs	Matrices	Reference
01	Jute	Polyester	[52]
02	Jute	Unsaturated Polyester	[53]
03	Jute	Polypropylene (PP)	[54]
04	Coir	Polyethylene (PE)	[55]
05	Hemp	PP	[56]
06	Hemp	Epoxy	[57]
07	Hemp	PP	[58,59]
08	Hemp	PLA	[60,61,62,63]
09	Wood Fiber	PE	[64]
10	Cellulose	PS and PP	[65,66]
11	Cellulose	Thermoplastic starch	[67]
12	Sisal	Epoxy	[2,68,69]
13	Flax	Epoxy	[70,71,72]
14	Flax	PP	[73,74,75]
15	Flax	Unsaturated Polyester	[53]
16	Kenaf	PLA	[76,77]
17	Kenaf	PP	[78]
18	Harakeke	Epoxy	[79,80]
19	Pineapple Leaf Fiber	Unsaturated polyester	[81]
20	Jute/Glass	Polyester (isophthalic)	[82,83]
21	Jute/Betel Nut	PP	[84]
22	Jute/Biomass	Bisphenol-C-formaldehyde resin	[85]
23	Jute/Cotton	Novolac phenolic	[86]
24	Jute/Glass	PP	[87]
25	Kenaf/Glass	Epoxy resin	[88]
26	Kenaf/Glass	Natural rubber	[89]
27	Rice Straw/Seaweed	PP	[90]
28	Betel Nuts/Seaweed	PP	[91]
29	Palmyra/Glass	Epoxy Resin	[92,93]
30	Bamboo/Glass	Vinyl Ester	[94]
31	Coir/Glass	Phenolic resin	[95]
32	Banana/Kenaf	Polyester	[96]
33	NF/Glass	Epoxy vinyl ester	[97]
34	Sisal/Kapok	Unsaturated polyester	[98]
35	Sisal/Glass	Unsaturated polyester	[99]
36	Sisal/Glass	Polyester	[100]
37	Sisal/Glass	Epoxy resin	[101,102]
38	Sisal/Glass	Phenolic	[103]
39	Sisal/Cotton	Polyester	[104]
40	Sisal/Silk	Unsaturated polyester	[105]
41	Oil Palm EFB/Jute	Epoxy resin	[106]
42	Cellulose/Glass	Epoxy resin	[107]
43	Flax/Glass	PP	[108]
44	Cotton/Silk	Polycarbonate (PC)	[109]
45	Wood Flour/Glass	Polyvinyl chloride (PVC)	[110]

**Table 3:** Classifications of plasmas [152], with permission from Wiley, 2017.

Plasma	State	Example
High-Temperature Plasma (Equilibrium Plasma)	T_e_ = T_i_ = T_h_ n_e_ ≥ 10^20^ m^−3^	Laser Fusion Plasma T_e_ = T_i_ = T_h_ ≈ 10^6^–10^8^ K
Low-Temperature Plasma	State	Example
Thermal Plasma (Quasi-Equilibrium Plasma)	T_e_ ≈ T_i_ ≈ T_h_ n_e_ ≥ 10^20^ m^−3^	Arc Plasma (core) T_e_ ≈ T_i_ ≈ T_h_ ≈ 10^4^ K
Non-Thermal Plasma (Non-Equilibrium Plasma)	T_e_ >> T_h_ N_e_ < 10^19^ m^−3^	Corona Discharge T_e_ ≈ 10^4^ – 10^5^ K T_h_ ≈ 3 × 10^2^−10^3^ K

Note: Te, Electron Temperature; Ti, Ion Temperature; Th, Heavy Particle Temperature; ne, Electron Density (state refers to the various parameters in the discharge process).

**Table 4:** Reported some studies of plasma-treated polymers for HM removal.

Sl. No.	Polymers/NFs	Plasma Treatment	Reference
1	Carboxymethyl cellulose carbon nanotube (CNT)	Nitrogen	[221]
2	CNT-Chitosan	Nitrogen	[222]
3	Reed-hemicellulose	Nitrogen	[223]
4	PET	Aam, AA, nitrogen	[210]
5	PS	Aam	[211]
6	PP-Chitosan	AA	[214]
7	PP	AA	[215]
8	PP	AA, oxygen	[219]
9	PES	Argon, oxygen	[205]
10	PE/PP	Argon	[218]
11	Chitosan	Glow discharge plasma	[224]
12	Polyvinylpyrrolidone (PVP)	Glow discharge plasma	[225]
13	Carbon	Atmospheric air	[226]
14	PP	Atmospheric air	[220]
15	Walnut shell	Water	[227]
16	Graphene	Allylamine	[228]
17	Silica-polymer	Thiophene	[206]
18	Cellulose (bamboo)	Epichlorohydrin, diethylenetriamine, and carbon disulfide	[229]

**Table 5:** Reported some studies of plasma-treated polymers for biomedical applications.

Sl. No.	NF/Polymer	Type of Plasma	Reference
1	PMMA	Oxygen	[230]
2	Poly (l-lactide-co-glycolide) (PLGA)	Oxygen	[244]
3	PLGA, Chitosan	Oxygen	[247]
4	PMMA	Oxygen	[251]
5	PP	Oxygen	[256]
6	PP	Oxygen	[266]
7	PP	Oxygen	[267]
8	Cyclo-olefin polymer wafer	Oxygen, C_4_F_8_	[231]
9	PMMA, PEEK	Oxygen, C_4_F_8_	[237]
10	PMMA, PS	Oxygen, C_4_F_8_	[235]
11	PMMA, PEEK, PDMS	Oxygen, SF_6_,	[232]
12	PET	Oxygen, Air	[257]
13	PP	Oxygen, Argon	[260]
14	PCL	Argon	[236]
15	PE, PLLA	Argon	[243]
16	poly-4-methyl-1-pentene	Argon	[248]
17	PLLA	Argon	[254]
18	PP	Argon	[263]
19	PE	Argon	[270]
20	PET, PP	Argon, Air, Helium	[241]
21	PLLA	Argon, Chitosan, Acetic acid	[242]
22	PP	Argon, Oxygen, polyphenylmethylsiloxyloxane	[258]
23	LDPE	Argon, acrylic acid polyethylene glycol	[271]
24	PLA	Argon, Air, Nitrogen, He	[245]
25	Poly urethane	Argon, NH_3_	[246]
26	Polyolefin plastomer	Allylamine, hexane	[249]
27	PTFE (Teflon)	Oxygen and methyl methacrylate	[233]
28	PP	Nitrogen	[238]
29	PP	Nitrogen, Propane, butane	[264]
30	PP	Air	[262]
31	PE	Air	[268]
32	PP	Bromine	[259]
33	LDPE	Acrylic acid	[269]
34	Polyolefin plastomer	Allylamine, hexane	[249]

**Table 6:** Reported some studies of plasma-treated polymers for water purification.

Sl. No.	NF/Polymer	Type of Plasma	Reference
1	poly (1,3-phenylene terephthalamide)	Oxygen, Argon	[276]
2	PP	Nitrogen	[277]
3	Almond shell	Nitrogen	[290]
4	PP	Carbon dioxide	[278]
5	PVDF	Carbon dioxide	[287]
6	Polyamide thin film composite	Hydrogen, Helium	[281]
7	Polyamide thin film composite	Hydrogen, Helium	[282]
8	PP	Air	[279]
9	Jatropha curcas shell	Humid air	[291]
10	cocoa shells	Humid air	[292]
11	PVDF	Carbon tetrafluoride	[288]
12	Chitosan	Glow discharge plasma	[286]
13	Thin film composite polyamide	Trimethylene glycol dimethyl ether	[280]

**Table 7:** Reported some studies of plasma-treated polymers for packaging applications.

Sl. No.	NF/Polymer	Type of Plasma	Reference
1	Zein	Atmospheric Air	[295]
2	polyethylene terephthalate/polypropylene	Atmospheric Air	[304]
3	Carboxymethyl cellulose-coated PP	Atmospheric Air	[317]
4	Chitosan	Atmospheric Air	[310]
5	Zein-chitosan	Atmospheric Air	[311]
6	Sodium caseinate film	Atmospheric Air	[318]
7	CSPCL	Atmospheric Air	[319]
8	Cassava starch films onto PCL and PLA	Atmospheric Air	[299]
9	Phlorotannin and Momordica charantia polysaccharides	Atmospheric Air	[320]
10	Corn starch	Air	[321]
11	Fish protein films	Dry Air	[322]
12	Whey and gluten proteins	Atmospheric Air and Argon	[323]
13	PLA	Air, Oxygen	[324]
14	PLA	Oxygen	[313]
15	PLA	Oxygen Air	[324]
16	Defatted soybean meal-based edible film	Oxygen. Nitrogen, Argon, Air, and He	[325]
17	PP	Oxygen, Hexamethyldisiloxane	[326]
18	Chitosan	Oxygen, Hexamethyldisilazane	[309]
19	Corn starch	Sulfur hexafluoride	[305]
20	Corn starch	Sulfur hexafluoride	[306]
21	Corn starch	hexamethyldisiloxane	[307]
22	Corn starch	hexamethyldisiloxane	[308]

**Table 8:** Reported some studies of plasma-treated polymers for sensing applications.

Sl. No.	Polymer/NF	Plasma	Sensing Parameter	Reference
1	PU/PDMS	Oxygen	Pressure	[327]
2	PDMS	Oxygen	Glucose	[343]
3	PAni/MWCNT	Oxygen	Ammonia	[348]
4	PEDOT: PSS	Oxygen	Gases	[338]
5	PEN	Oxygen	Temperature	[332]
6	GWF/PDMS/cellulose	Oxygen	Temperature, Humidity	[334]
7	PMMA	Argon	Humidity	[333]
8	PAni/MWCNT/l-carrageenan	Argon	Hydrogen	[339]
9	Silicon/functionalized polymer	Oxygen/N_2_H_2_/CF_4_	Humidity	[349]
10	PS, PVP, PCL, PP, PE	Atmospheric air	Dopamine	[344]
11	LDPE	Atmospheric air	Glucose and Dopamine	[345]
12	Poly(para-xylylene)	Atmospheric air	Human Hepatitis B	[350]
13	PEDOT: PSS	Nitrogen	Pressure	[328]
14	Carbon electrode	Allylamine	Phenolic components	[351]

## Glossary

NF	Natural fiber
PSM	Plasma surface modification
NFC	Natural fiber composite
NFRPC	Natural fiber reinforced polymer composite
LTP	low-temperature/cold plasma
MRE	metal removal efficiency
HM	heavy metal
SEM	scanning electron microscopy
XPS	X-ray photoelectron spectroscopy
FTIR	Fourier transform infrared spectroscopy
AAS	atomic absorption spectrophotometry
ES	electrospun
PE	polyethylene
PP	polypropylene
PS	polystyrene
PLA	polylactic acid
PLLA	poly-l-lactic acid
PCL	polycaprolactone
PET	polyethylene terephthalate
PEN	polyethylene naphthalate
PC	polycarbonate
PVC	polyvinyl chloride
PES	polyether sulfone
Aam	acrylamide
AA	acrylic acid
PEG	polyethylene glycol
PU	polyurethane
RO	reverse osmosis
PSS	polystyrene sulfonate
MMA	methyl methacrylate
PMMA	poly methyl methacrylate
VP	vinyl pyrrolidone
PVP	polyvinyl pyrrolidone
MWCNT	multiwalled carbon nanotube
LDPE	low-density polyethylene
HDPE	high-density polyethylene
CNT	carbon nanotube
PEEK	polyether ether ketone
VBAC	(vinyl benzyl) trimethylammonium chloride
UHMWPE	ultra-high molecular weight polyethylene
